# A Danish questionnaire study of acute symptoms of SARS-CoV-2 infection by variant, vaccination status, sex and age

**DOI:** 10.1038/s41598-023-47273-8

**Published:** 2023-11-14

**Authors:** Anna Irene Vedel Sørensen, Lampros Spiliopoulos, Peter Bager, Nete Munk Nielsen, Jørgen Vinsløv Hansen, Anders Koch, Inger Kristine Meder, Anders Hviid, Steen Ethelberg

**Affiliations:** 1 Infectious Disease Epidemiology and Prevention, Statens Serum Institut, 2300 Copenhagen S, Denmark; 2 Department of Epidemiology Research, Statens Serum Institut, 2300 Copenhagen S, Denmark; 3 Focused Research Unit in Neurology, Department of Neurology, Hospital of Southern Jutland, University of Southern Denmark, 6200 Aabenraa, Denmark; 4 Department of Infectious Diseases, Rigshospitalet University Hospital, 2100 Copenhagen Ø, Denmark; 5 Department of Public Health, Global Health Section, University of Copenhagen, 1014 Copenhagen K, Denmark; 6 Pharmacovigilance Research Centre, Department of Drug Design and Pharmacology, University of Copenhagen, 2100 Copenhagen Ø, Denmark

**Keywords:** Viral infection, Signs and symptoms

## Abstract

It is not well-described how the acute symptoms of infection with severe acute respiratory syndrome coronavirus 2 (SARS-CoV-2) differ by variant, vaccination, sex and age. A cross-sectional questionnaire study linked to national testing- and registry data was conducted among 148,874 SARS-CoV-2 first time reverse transcription polymerase chain reaction (RT-PCR) test-positive individuals and corresponding date-matched symptomatic test-negative controls. Major SARS-CoV-2 variants (Index/wild type, Alpha, Delta and Omicron) were defined using periods of predominance. Risk differences (RDs) were estimated for each of 21 predefined acute symptoms comparing: (1) test-positive and -negative individuals, by variant period, (2) vaccinated and unvaccinated test-positives, by variant period, (3) individuals tested positive during the Omicron and Delta periods, by vaccination status, and (4) vaccinated Omicron test-positive and -negative individuals, by age and sex. Compared to pre-Omicron, RDs between test-positive and test-negative individuals during the Omicron period were lower for most symptoms. RDs for altered sense of smell (dysosmia) and taste (dysgeusia) were highest for Delta (RD = 50.8 (49.4–52.0) and RD = 54.7 (53.4–56.0), respectively) and lowest for Omicron (RD = 12.8 (12.1–13.5) and RD = 11.8 (11.1–12.4), respectively). Across variants, vaccinated individuals reported fewer symptoms. During Omicron, females and 30–59 year-old participants reported more symptoms.

## Introduction

Presentations of SARS-CoV-2 infection ranges from asymptomatic carriage to fatal COVID-19. In the pre-Omicron variant period of the pandemic, acute respiratory manifestations together with altered sense of smell (dysosmia) and taste (dysgeusia) were the most distinctive features of symptomatic infection^1,2^. Relative to previous variants, the Omicron variant and its sub-variants causes less severe disease in the form of reduced risks of hospitalization, intensive care unit stay and death^3,4^. Some studies of differences between symptoms caused by different variants of SARS-CoV-2 already exist^4–6^. One study has shown that the mean number of symptoms caused by Omicron were lower than for Delta^6^, whereas other studies have found different results^7^. Compared to infection with pre-Omicron variants, changes in sense of smell (dysosmia) and taste (dysgeusia) seem to be less common during infection with the Omicron variant^4–6,8,9^. Several studies also reported that sore throat was more common during the Omicron period^4–6,8^.

Soon after the emergence of the Omicron variant, it became clear that the original vaccines did only provide limited protection against infection with this variant^10–12^. Vaccination still reduced the severity of disease^13^, however less is known about the effect on the symptom profile among those suffering from mild or moderate disease. The results of one study indicate that vaccination is associated with reduced odds of reporting more than five symptoms within the first week of illness, as well as reduced odds of extended duration of symptoms (≥ 28 days)^14^.

The COVID-19 surveillance results in many countries suggest that being female and of younger age reduces the severity of infection^15^, although the underlying causes are not well understood. Other potential impacts of age and sex on acute symptomatology are not entirely clear.

Many of the previous studies of clinical manifestations of infection with SARS-CoV-2 have focused on severe consequences of COVID-19 such as hospitalizations and deaths. However, given their impact in numbers, it is essential to describe the acute symptoms of SARS-CoV-2 infection experienced by the vast majority of COVID-19 cases, i.e. the non-hospitalized cases, and clarify how these symptoms are modified by different factors.

In the present study, we took advantage of a large nationwide questionnaire study on acute- and post-acute symptoms of SARS-CoV-2 infection in the adult Danish population to evaluate the importance of SARS-CoV-2 variants stratified by vaccination status, sex and age. Main emphasis was on the Omicron variant.

## Results

### Participants

During the period September 1, 2021 to March 16, 2022, we invited 2,154,924 individuals to complete the questionnaire. Following the exclusion of 640,438 individuals whose test date fell in between the four periods of variant predominance, 1,514,486 individuals (41.5% test-positive) were eligible for inclusion (Supplementary Fig. S1).

Among these, 468,286 (30.9%) participants completed the questionnaire, whereas 40,107 (2.7%) partially filled-out the questionnaire and 1,006,093 (66.4%) did not respond, and were thus excluded from the study. The resulting response rates were 27.7% and 33.2% for the invited 627,825 test-positives and 886,661 test-negatives, respectively. Among the 468,286 eligible participants, only the 29,227 test-negatives with symptoms as test indication were included in analysis, leading to exclusion of 264,995 test-negatives. Among the remaining test-negatives, a group of 5105 reported being seropositive before replying to the questionnaire and were therefore excluded. Furthermore, 2329 participants who were vaccinated less than fourteen days before the test date, 4920 participants vaccinated with first dose only, and 42,063 participants who were vaccinated more than four months and two weeks prior to the test, were also excluded.

Following the application of the aforementioned exclusion criteria, the final study population consisted of 148,874 participants (130,202 test-positives and 18,672 test-negatives) (Supplementary Fig. S1).

### Characteristics of the participants

The final 148,874 participants consisted of 90,627 females (60.9%) and 58,247 males (39.1%) with median ages of 48 years (IQRs: 34, 59) and 52 years (IQRs: 39, 62), respectively (Table S1). The distribution of participants within variant periods were: Index: 47,605 (92.7% positive); Alpha: 16,249 (94.2% positive); Delta: 20,095 (81.8% positive) and Omicron: 64,925 (83.6% positive). Across all periods, at least one comorbidity was reported by 34.8% of participants.

### Comparison of acute symptoms among test-positive and symptomatic test-negative participants, stratified by variant period

**Figure 1:**
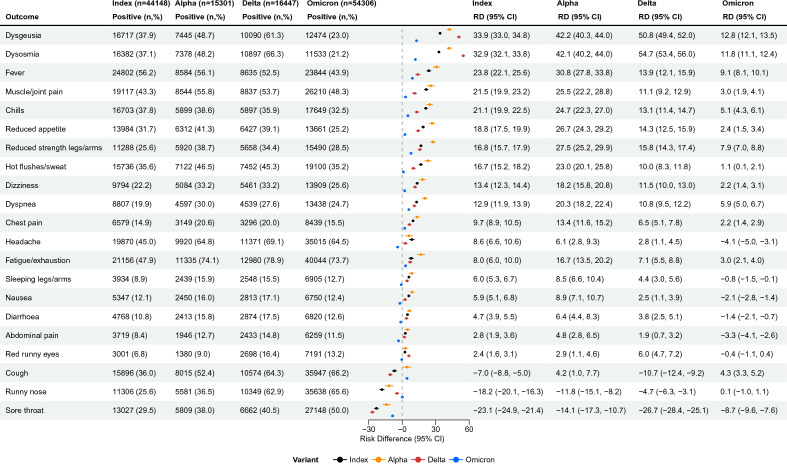
Acute symptomatology: Risk-differences between test-positives and symptomatic test-negatives for self-reported acute symptoms, stratified by variant, N = 148.874, Denmark. *n* number of participants, *RD* Risk difference, *CI* Confidence interval.

All symptoms, except for runny nose and sore throat, were more often reported by test-positive than test-negative participants (Fig. 1, Supplementary Table S2). During the Omicron period, most symptoms had lower RDs than in other variant periods, and several symptoms, such as headache (RD =  − 4.1%, 95% CI − 5.0 to − 3.1), nausea (RD =  − 2.1%, 95% CI − 2.8 to − 1.4), diarrhoea (RD =  − 1.4%, 95% CI − 2.1 to − 0.7) and abdominal pain (RD =  − 3.3%, 95% CI − 4.1 to − 2.6) were more common among the test-negative participants than among the participants tested positive in the Omicron period (Fig. 1, Supplementary Table S2).

Across all variant periods, the symptoms with the largest RDs were dysgeusia and dysosmia, although with comparatively lower RDs during the Omicron period (RD = 12.8%, 95% CI 12.1–13.5 and 11.8%, 95% CI 11.1–12.4, respectively) compared to other periods (Index: dysgeusia: 33.9%, 95% CI 33.0–34.8, dysosmia: 32.9%, 95% CI 32.1–33.8; Alpha: dysgeusia: 42.2%, 95% CI 40.3–44.0, dysosmia: 42.1%, 95% CI 40.2–44.0; Delta: dysgeusia: 50.8%, 95% CI 49.4–52.0, dysosmia: 54.7%, 95% CI 53.4–56.0) (Fig. 1, Supplementary Table S2). The RDs for fever and dyspnea, which could indicate more severe disease, were highest during the Alpha period (RD = 30.8%, 95% CI 27.8–33.8 and RD = 20.3%, 95% CI 18.2–22.4, respectively) and lowest during the Omicron period (RD = 9.1%, 95% CI: 8.1–10.1 and RD = 5.9%, 95% CI 5.0–6.7, respectively).

### Comparison of acute symptoms among fully vaccinated and unvaccinated test-positive individuals during three different variant periods

Due to the very limited number of individuals vaccinated during the period dominated by the Index variant, this variant period was not included in this comparison between fully vaccinated and unvaccinated participants.

Among fully vaccinated test-positives, 93.5% reported at least one symptom compared to 92.6% among unvaccinated test-positives with median (IQR) numbers of symptoms equal to 7 (4, 11) and 9 (5, 12), respectively.

**Figure 2:**
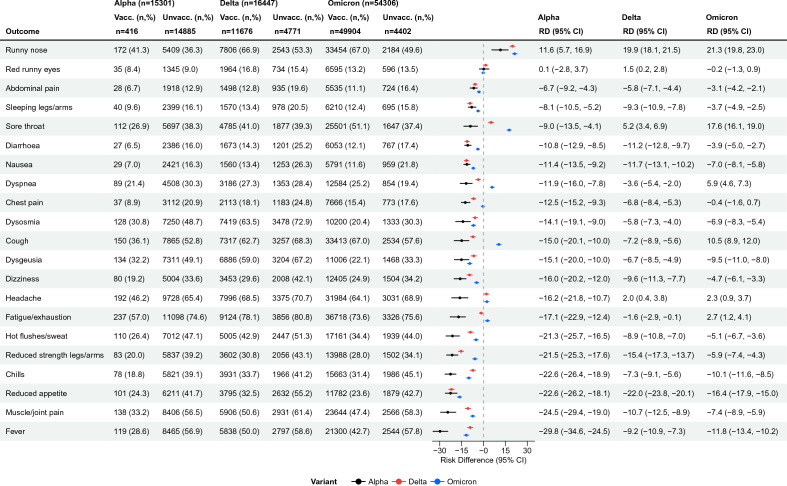
Acute symptomatology across vaccination status: Risk-differences between fully vaccinated and unvaccinated test-positives, stratified by variant, N = 86,054, Denmark. *Vacc.* Vaccinated, *Unvacc.* Unvaccinated, *n* number of participants, *RD* Risk difference, *CI* Confidence interval.

Across all three variant periods, especially in the Alpha period, most symptoms were less often reported by vaccinated individuals. However, during the Omicron period, runny nose (RD = 21.3%, 95% CI 19.8–23.0), sore throat (RD = 17.6%, 95% CI 16.1–19.0), cough (RD = 10.5%, 95% CI 8.9–12.0), dyspnea (RD = 5.9%, 95% CI 4.6–7.3), fatigue/exhaustion (RD = 2.7%, 95% CI 1.2–4.1) and headache (RD = 2.3%. 95% CI 0.9–3.7) were more often reported by vaccinated than unvaccinated individuals (Fig. 2).

### Comparison of acute symptoms among test-positive participants during the Omicron and Delta periods, stratified by vaccination status

For participants having recently (within 4 months) received a full vaccination course consisting of two doses and no additional boosters, the median time intervals between the second dose and the test date were 3.2 months (Delta) and 3.7 months (Omicron). The corresponding time intervals for participants who had recently (within 4 months) received a booster were 0.9 (Delta) and 1.1 months (Omicron).

**Figure 3:**
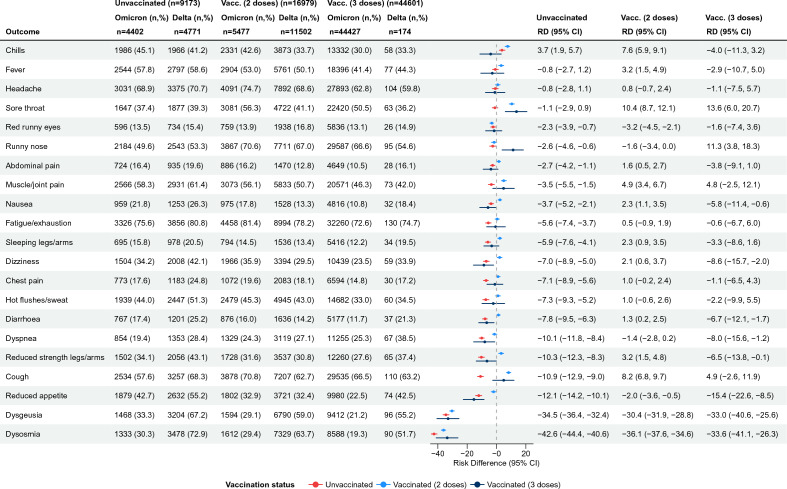
Acute symptomatology in relation to vaccination status: Risk differences between test-positives infected during Omicron and Delta periods (reference), stratified by vaccination status, N = 70,753, Denmark. *Vacc.* Vaccinated, *n* number of participants, *RD* Risk difference, *CI* Confidence interval.

Among test-positive unvaccinated participants, most symptoms were less often reported during the Omicron period compared to the Delta period, apart from chills (RD = 3.7%, 95% CI 1.9–5.7). Among participants vaccinated with two doses, eleven out of twenty-one symptoms were more often reported during the Omicron than Delta period with the largest differences observed for sore throat (RD = 10.4%, 95% CI 8.7–12.1), cough (RD = 8.2%, 95% CI 6.8–9.7) and chills (RD = 7.6%, 95% CI 5.9–9.1) (Fig. 3). For participants, who had received a booster, only two symptoms were more prevalent during the Omicron than Delta period; namely sore throat (RD = 13.6%, 95% CI 6.0–20.7) and runny nose (RD = 11.3%, 95% CI 3.8–18.3) (Fig. 3).

### Comparison of acute symptoms reported by fully vaccinated test-positives and fully vaccinated symptomatic test-negatives during the Omicron period, stratified by age and sex

**Figure 4:**
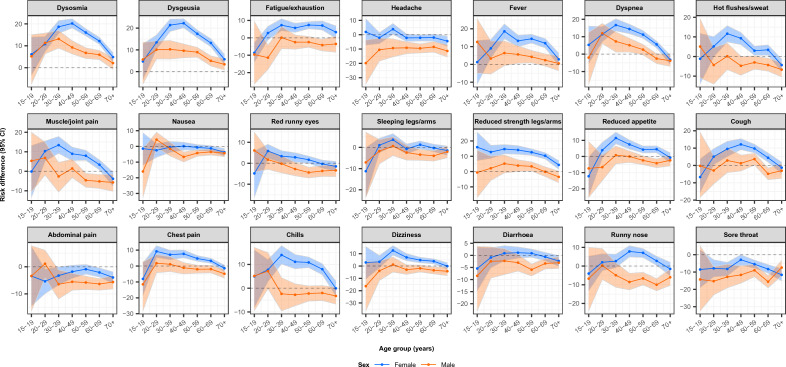
Acute symptomatology during the Omicron period: Risk differences between vaccinated test-positives and -negatives with symptoms as test indication, stratified by sex and age, N = 60,241, Denmark. The bands on the figure indicate 95% confidence intervals for the risk difference estimates.

The majority of symptoms tended to be more often reported by females, and especially by 30–59 years old participants (Fig. 4, Supplementary Table S3). RDs for experiencing at least one of the symptoms dysosmia, dysgeusia and fever, were higher for females (RD = 17.5%, 95% CI 16.2–18.7) than males (RD = 6.4%, 95% CI 4.6–8.2). Additionally, RDs for experiencing at least one of these symptoms were higher for 30–59 years old participants (RD = 17.7%, 95% CI 16.2–19.1) compared to other age groups (15–29 and 60 +) (RD = 9.4%, 95% CI 7.9–10.9).

When combining sex and age groups, the largest risk differences for experiencing at least one of the symptoms dysosmia, dysgeusia and fever were observed for 30–59 years old females (RD = 21.7%, 95% CI 20.0–23.6), compared to the combined group of 15–29 or 60 + years old females (RD = 12.4%, 95% CI 10.2–14.4), 30–59 years old males (RD = 9.0%, 95% CI 6.3–11.7), and 15–29 or 60 + years old males (RD = 2.2%, 95% CI − 0.4 to 4.7).

### Acute symptoms reported by fully vaccinated and unvaccinated test-positives infected during the Omicron period, stratified by time since the most recent vaccine dose (second or third)

To assess the potential influence of only including persons where less than 4 months and 14 days had elapsed since last vaccination in the main analyses, a sub-analysis of risk-differences between vaccinated and unvaccinated participants was conducted, where vaccinated persons for whom more time had elapsed since vaccination were included as a separate group (Supplementary Fig. S2). For the majority of the 21 symptoms included, we did not observe any significant differences between the risk differences for those vaccinated within 4 months and 14 days and those were more time had elapsed. For those symptoms where significant differences were observed, the general trends were more pronounced for the recently vaccinated group, i.e. for runny nose and sore throat, where the risk differences between vaccinated and unvaccinated participants were positive, these were greater for those where less time had elapsed since vaccination, whereas for chills, fever and reduced appetite where the risk differences between vaccinated and unvaccinated participants were negative, these were lower for the more recently vaccinated group.

## Discussion

By using a very large data material of questionnaire responses from the nationwide EFTER-COVID cohort, we found that for most symptoms the risk differences between test-positive and test-negative participants were lower during the Omicron than pre-Omicron period. These differences were most notable for dysosmia and dysgeusia.

Across all variants*,* except for the Index variant where not enough post-vaccination data were available to evaluate this, recent full vaccination provided some protection against most covid-19 related symptoms, such as fever, dysgeusia and dysosmia, but not necessarily against cold-like symptoms, such as runny nose and sore throat.

During the Omicron period, more than half of the 21 symptoms included in this survey were less prevalent among participants recently vaccinated with three doses, compared to those who had only received two doses. The median time since receiving the last dose was lower for those who had received a third dose compared to those who had only received two. Therefore, it is not known whether the observed differences in the symptom pattern are mainly caused by the number of doses or the time between the most recent vaccination and testing.

The majority of symptoms were more often reported by females than males, and mostly among those aged 30–59.

The lower prevalences of dysosmia or dysgeusia among individuals infected by the Omicron variant have also been observed in other studies^4–6,8,9^, albeit the reported prevalences vary considerably (range for dys-/anomsia: 5–20%)^4–6,8,9^. During the Omicron period, gastrointestinal symptoms were more often reported by our test-negative participants, compared to the test-positive participants. Similar results were obtained in another study^5^, where it was suggested that the decline in gastrointestinal symptoms compared to pre-Omicron could be linked to in vitro findings of reduced replication of Omicron in intestinal cells^16^.

For the most frequently used vaccines in Denmark, BNT162b2 (Pfizer) and mRNA-1273 (Moderna), the estimated vaccine effectiveness against developing symptomatic infection with the Omicron variant (39.9% for > 60-year old participants, 14–30 days after vaccination) was markedly lower than for the Delta (82.2%) and the Alpha variant (91.0%)^13^. Protection against symptomatic Omicron infection decreased to 4.7% after > 120 days^13^, which was one of the main arguments for only including recently vaccinated (within 4 months) in the present study. Estimates for vaccine effectiveness were higher (57.6%) 14–30 days after the third dose^13^. In other studies, vaccination has been associated with shorter duration of disease and lower risk of experiencing more than five symptoms^14^, and especially lower risk of loss of taste or smell^6^. Our observations confirmed that vaccinated individuals tended to report fewer symptoms than unvaccinated, but for the Omicron variant, some symptoms were more often reported by vaccinated individuals. A subanalysis showed that these effects were still observable for those where more time had elapsed since vaccination. It is not clear why some symptoms were more often reported by vaccinated individuals, however receiving a booster dose, did seem to have an effect on the occurrence of most symptoms. Thus, the decision to provide a booster dose after the emergence of the Omicron variant might not only have reduced hospitalization rates, but also the number of symptoms experienced by those suffering from milder disease.

Despite less mortality and lower hospitalization rates for females in other studies, the majority of symptoms in our study were more often reported by females than males. In general, the higher occurrence of severe COVID-19 among males than females has been attributed to differences in the prevalence of comorbidities, as well as sex-specific biological differences^17^. Albeit also observed in other studies^18,19^, it is not clear why females more frequently report certain symptoms, especially disturbances in smell and taste. However this phenomenon has also been observed for other infections^20^.

We observed an influence of age with higher RDs among the 30–59 years old participants. Similar results with fewer symptoms reported by the young and the elderly, and a pronounced difference between age groups for dysosmia, have been reported by others^6^. In general, non-COVID-19-related reduced sense of smell has been reported to affect more than half of those 65–80 years old^21^, so it could be hypothesized, that this group might be less likely to notice any COVID-19 related changes.

The major strengths of the present study are the large study population, and the use of date-matched control groups for each variant period.

The primary limitations of the study are the self-reporting of symptoms and the response rate. The motivation for participation might be lower among less symptomatic individuals and those experiencing very few symptoms only, potentially leading to selection bias. However, response rates were higher among test-negatives than test-positives, which supports the validity of our results.

Due to varying time intervals between the test date and the distribution of the questionnaire, any potential recall bias might be higher during the Index and Alpha periods, compared to the Delta and Omicron periods, and this might potentially be larger for test-negative participants, since they might have paid less attention to their well-being. However, in Fig. 1 we reported risk differences between test-positive and test-negative participants within the same periods, and since these were date-matched, the effect of recall bias on estimated differences between variants is expected to be limited.

Another limitation of the study is that it was not possible to compare subvariants of Omicron. Results of another study have indicated that the symptoms profiles of BA.1 and BA.2 might differ^6^. Additionally, we might have failed to take into account the potential influence of comorbidities not included in the questionnaire.

## Conclusion

Infection with the Omicron variant less often caused dysgeusia and dysosmia. Across variants, vaccinated individuals reported fewer symptoms. During Omicron, females and 30–59 year-old participants reported more symptoms than other population groups.

Awareness about differences in symptoms due to SARS-CoV-2 variants and vaccination status will be helpful for clinicians and public health officials when considering future vaccination and test recommendations.

## Methods

### Study design

In Denmark, free-of-charge access to reverse transcription polymerase chain reaction (RT-PCR) tests for SARS-CoV-2 independent of test indication has been widely accessible since May 2020^22^. During the pandemic, the test incidences in Denmark have been among the highest in the world^23^, with RT-PCR test-incidences of up to ~ 27,360 per 100,000 inhabitants and total test-incidences, including antigen tests, of up to ~ 73,100 per 100,000 inhabitants during the study period^24^.

Based on records in the national COVID-19 surveillance system at Statens Serum Institut, which captures the individual results of all SARS-CoV-2 RT-PCR tests performed in Denmark, participants were invited to take part in a national survey of post-acute symptoms, EFTER-COVID. In this survey all baseline questionnaires also included information about acute symptoms used for the present study.

All individuals, who tested positive for the first time within the study period and had an account in the national digital post system, were invited to participate in the study. Controls were selected using incidence density sampling among all test-negatives on a given date, who had a digital post account and did not have any positive test records. Based on lower expectations for the response rate among test-negatives, a test-positive to test-negative ratio of 2:3 was used.

Data were collected for test-dates between September 1, 2020 and February 16, 2022. According to test date, participants were attributed to different variants of SARS-CoV-2, based on when each variant was predominant in Denmark (for information about the evolution of the pandemic in Denmark, please see Fig. 1 in Michlmayr et al.^25^): Index (wild type) (1 August 2020–31 December 2020); Alpha (15 March–30 June, 2021); Delta (15 July–15 November, 2021); and Omicron (28 December, 2021–16 February, 2022). Test-positive and -negative individuals whose test date fell within the intermediate transitional periods were excluded. Thus, the sample size was determined by the number of individuals receiving a positive test result during the periods specified for each variant.

Self-reported symptom data were collected using a web-based questionnaire, distributed through the national digital post system, eBoks. Use of eBoks is mandatory for all inhabitants in Denmark aged 15-years or above, unless exempted due to disabilities/functional impairment or practicalities e.g. lack of internet access.

Participants received the invitation to participate either 9 or 12 months (Index period), or 2, 4 or 6 months (Alpha period) or 30 days (Delta and Omicron periods) after their test date.

To reduce misclassification bias, individuals full-filling one of the following criteria were excluded from the main study: (1) Test-negative participants, who had been tested for other reasons than experiencing symptoms, (2) Test-negative persons, who reported testing seropositive for SARS-CoV-2 prior to invitation; (3) Partially vaccinated persons, who did not receive the full primary vaccination course, but only first dose; (4) Persons, who finished vaccination within the last 14 days before the test date, where vaccination might not have yet reached full effect; (5) Vaccinated persons, where more than 4 months and 14 days had elapsed since last vaccination at the test date and therefore the protective effect against symptomatic disease might have waned considerably (time interval selected based on data from the present study). Persons, where more than 4 months and 14 days had elapsed since last vaccination at the test date were included in a sensitivity analysis (Supplementary Fig. S2).

The questionnaire contained questions about whether respondents had experienced any of 21 specific symptoms during the period from one week before and until four weeks after the test date. In addition, the questionnaire collected information about e.g. education, employment, height, weight, life style and certain comorbidities. Furthermore, test-negatives were asked about test indication and whether they suspected ever having had COVID-19, and whether they had ever been tested seropositive.

To avoid information bias due to test-positive participants omitting non-COVID-19 symptoms, while test-negative participants still reported these, participants were specifically asked to report any symptom that they might have experienced, no matter the perceived cause.

The questionnaire data was supplemented with information derived from various registers: age and sex from the Danish civil registration system, health care occupation (yes/no) from the COVID-19 surveillance database, and diagnoses from the Danish National Patient Register (DNPR). The DNPR contains information on in- and outpatient diagnoses in the hospital setting coded using the International Classification of Diseases 10th revision (ICD-10), which enabled calculation of Charlson Comorbidity Index scores^26^. Data for SARS-CoV-2 vaccination status of participants were obtained from the Danish Vaccination Register. In Denmark, the vaccines used are (based on last dose of the primary courses given until February 16, 2022): BNT162b2 (Pfizer/BioNTech) 85.9%, mRNA-1273 (Moderna) 13.0%, ChAdOx1-S (AstraZeneca) 1%, and Ad26.COV2.S (Janssen) < 0.1%^27^. From late 2021 and onwards, a booster dose was offered to those above 18 years.

### Statistical analysis

The prevalence of symptoms in groups characterized by test-status, variant, vaccination status, age and sex, were compared using risk differences (RDs). RDs including 95% confidence intervals were estimated using parametric g-computation^28^ on logistic regression. The 95% confidence intervals were estimated through bootstrap random resampling with 1000 iterations. We estimated RDs for each of the 21 predefined acute symptoms comparing: (1) test-positives and test–negatives (reporting symptoms as test indication), by variant period, (2) fully vaccinated (two or three doses) and unvaccinated test-positives, by variant period, (3) individuals tested positive during the Omicron and Delta periods, respectively, by vaccination status (unvaccinated, full course (two doses) or booster received (three doses)), and (4) fully-vaccinated (two or three doses) test-positive and test-negative individuals during the Omicron period, by age and sex.

The estimates were adjusted for BMI category, self-reported comorbidities from the questionnaire, Charlson Comorbidity Index scores, healthcare occupation, as well as age, sex, and vaccination status when applicable. Based on results from other studies, these variables were á priori considered potential confounders in relation to acute symptoms.

Charlson Comorbidity Index scores^26^ were calculated based on data for the past five years extracted from the DNPR. Since few scores were above 3, these were included in analyses as 0, 1, 2 or ≥ 3. In addition, the questionnaire contained questions about comorbidities commonly treated in primary care (Table S1) and therefore unlikely to be listed in the DNPR. The presence of these comorbidities was included in analyses as a dichotomous variable. For individuals aged 18 years or above, obesity was defined as BMI ≥ 30, while international cut-off points by sex and age^29^ were used for 15–17 year old participants.

Only data from completed questionnaires were included in the analyses. All questions were mandatory, except those on height, weight, smoking and alcohol consumption. When adjusting for BMI category, missing data for height or weight were handled by creating the category “unknown”.

Data management and statistical analyses were conducted using R version 4.1.3^30^. The R-packages “riskCommunicator”^31^ and “forestploter”^32^ were used for modelling and generation of forest plots, respectively.

### Ethics approval and informed consent

This article has been prepared on the basis of a study carried out as part of a task imposed on the Statens Serum Institut (SSI) according to national legislation. Therefore, no approval requirements from the ethics committees is obliged. All methods were carried out in accordance with relevant guidelines and regulations.

The publication only contains aggregated results and no personal data. The publication is therefore not covered by the European General Data Protection Regulation. It was approved by the Danish Governmental law firm (“Kammeradvokaten”) and SSI’s Compliance Department (“Data protection and Information Security”) that the study is fully compliant with all legal, ethical and IT-security requirements and there are no further approval procedures regarding such studies. Thus, no further approval of the experimental protocol was needed.

Participation in the study was voluntary. The invitation letter to participants contained information about their rights under the Danish General Data Protection Regulation (rights to access data, rectification, deletion, restriction of processing and objection). Accessing and filling in the questionnaire after receiving the above information was considered informed consent from the participant’s side. Therefore informed consent was obtained from all subjects and/or their legal guardians.

### Supplementary Information

Supplementary Figures.

Supplementary Table S1.

Supplementary Table S2.

Supplementary Table S3.

## Data Availability

The datasets used in the study comprises individual-level sensitive information from completed questionnaires and national register data. According to the Danish data protection legislation, the authors are not allowed to share these sensitive data directly upon request. However, the data are available for research upon reasonable request to The Danish Health Data Authority (register-data, e-mail: kontakt@sundhedsdata.dk) and Statens Serum Institut (questionnaire data, e-mail: aii@ssi.dk) and within the framework of the Danish data protection legislation and any required permission from Authorities. Expect a time-frame of at least 3 to 6 months for data requests to be processed.

## Abbreviations

BMI: Body mass index
CI: Confidence interval
DNPR: Danish National Patient Register
ICD-10: International classification of diseases 10th revision
IQR: Interquartile range
RD: Risk difference
RT-PCR: Reverse transcription polymerase chain reaction
SARS-CoV-2: Severe acute respiratory syndrome coronavirus 2
