# Supplementary material for: A Danish questionnaire study of acute symptoms of SARS-CoV-2 infection by variant, vaccination status, sex and age
Source: Sci Rep. 2023 Nov 14;13:19863. doi: 10.1038/s41598-023-47273-8 (PMC10645837; doi:10.1038/s41598-023-47273-8)
Supplement: Supplementary file 1 — Supplementary Figures. [file 41598_2023_47273_MOESM1_ESM.pdf]

Supplementary Information

Sørensen AIV, Spiliopoulos L, Bager P, Nielsen NM, Hansen JV, Koch A, Meder IK, Hviid A & Ethelberg, S (2023): A Danish questionnaire study of acute symptoms of SARS-CoV-2 infection by variant, vaccination status, sex and age

Figure S1: Flowchart for selection of study population

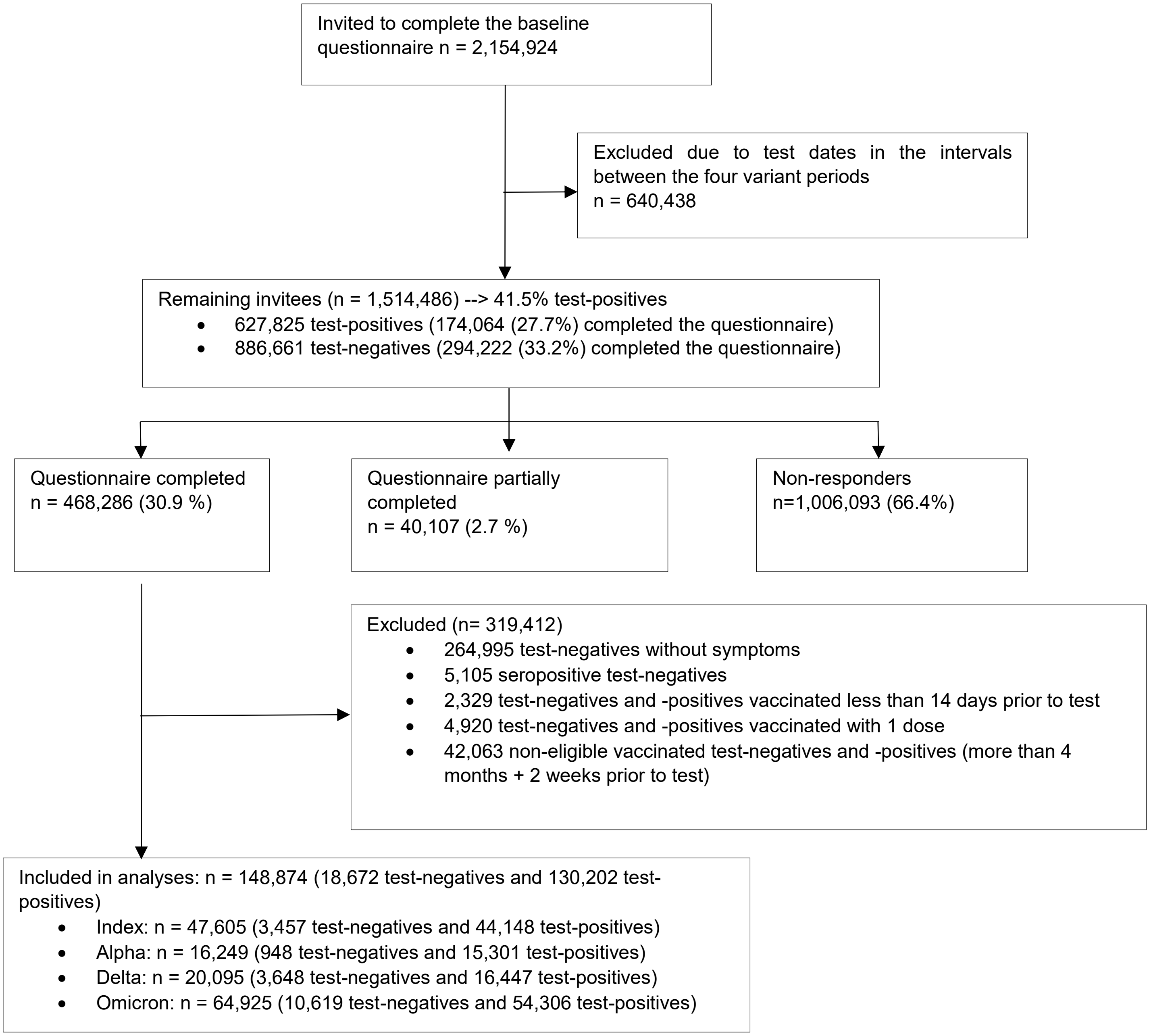

Figure S2: Acute symptomatology in relation to vaccination status: Risk differences between fully vaccinated and unvaccinated test−positives infected during Omicron, stratified by the time since the most recent second or third COVID−19 vaccine dose, N=84,447, Denmark.

Outcome	<4.5 months Vacc. (n,%) n=49904	>4.5 months Vacc. (n,%) n=30141	Unvacc. (n,%) n=4402	Vacc. (<4.5 months) RD (95% CI)	Vacc. (>4.5 months) RD (95% CI)
Runny nose	33454 (67.0)	19801 (65.7)	2184 (49.6)	21.3 (19.8, 23.0)	15.7 (14.1, 17.0)
Sore throat	25501 (51.1)	15662 (52.0)	1647 (37.4)	17.6 (16.1, 19.0)	14.3 (12.8, 15.8)
Cough	33413 (67.0)	20256 (67.2)	2534 (57.6)	10.5 (8.9, 12.0)	9.5 (8.0, 11.2)
Dyspnea	12584 (25.2)	7558 (25.1)	854 (19.4)	5.9 (4.6, 7.3)	6.2 (4.9, 7.5)
Fatigue/exhaustion	36718 (73.6)	23637 (78.4)	3326 (75.6)	2.7 (1.2, 4.1)	2.9 (1.6, 4.3)
Headache	31984 (64.1)	21633 (71.8)	3031 (68.9)	2.3 (0.9, 3.7)	2.6 (1.1, 4.0)
Red runny eyes	6595 (13.2)	3993 (13.2)	596 (13.5)	−0.2 (−1.3, 0.9)	−0.5 (−1.8, 0.5)
Chest pain	7666 (15.4)	5554 (18.4)	773 (17.6)	−0.4 (−1.6, 0.7)	1.0 (−0.2, 2.2)
Abdominal pain	5535 (11.1)	4432 (14.7)	724 (16.4)	−3.1 (−4.2, −2.1)	−2.4 (−3.6, −1.2)
Sleeping legs/arms	6210 (12.4)	4209 (14.0)	695 (15.8)	−3.7 (−4.9, −2.5)	−1.8 (−3.0, −0.6)
Diarrhoea	6053 (12.1)	4270 (14.2)	767 (17.4)	−3.9 (−5.0, −2.7)	−2.9 (−4.1, −1.8)
Dizziness	12405 (24.9)	10009 (33.2)	1504 (34.2)	−4.7 (−6.1, −3.3)	−2.0 (−3.5, −0.5)
Hot flushes/sweat	17161 (34.4)	12752 (42.3)	1939 (44.0)	−5.1 (−6.7, −3.6)	−2.1 (−3.5, −0.4)
Reduced strength legs/arms	13988 (28.0)	9338 (31.0)	1502 (34.1)	−5.9 (−7.4, −4.3)	−3.1 (−4.7, −1.7)
Dysosmia	10200 (20.4)	7152 (23.7)	1333 (30.3)	−6.9 (−8.3, −5.4)	−5.8 (−7.2, −4.5)
Nausea	5791 (11.6)	5119 (17.0)	959 (21.8)	−7.0 (−8.1, −5.8)	−5.2 (−6.6, −3.9)
Muscle/joint pain	23644 (47.4)	15680 (52.0)	2566 (58.3)	−7.4 (−8.9, −5.9)	−4.6 (−6.1, −3.0)
Dysgeusia	11006 (22.1)	7319 (24.3)	1468 (33.3)	−9.5 (−11.0, −8.0)	−8.0 (−9.6, −6.7)
Chills	15663 (31.4)	11517 (38.2)	1986 (45.1)	−10.1 (−11.6, −8.5)	−6.5 (−8.2, −4.9)
Fever	21300 (42.7)	14739 (48.9)	2544 (57.8)	−11.8 (−13.4, −10.2)	−8.4 (−10.1, −6.8)
Reduced appetite	11782 (23.6)	9497 (31.5)	1879 (42.7)	−16.4 (−17.9, −15.0)	−12.0 (−13.6, −10.4)

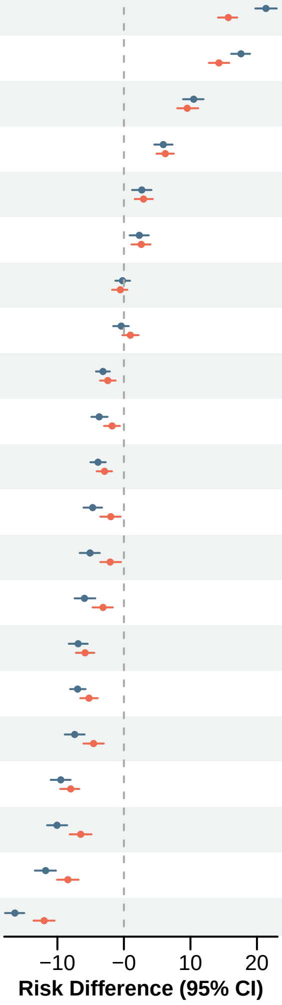

Time since the most recent second or third COVID−19 vaccine dose (in months) <4.5 >4.5
